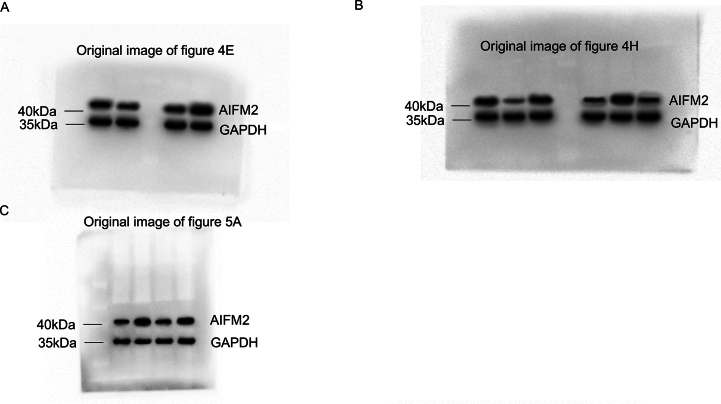# Long noncoding RNA ZFAS1 exerts a suppressive impact on ferroptosis by modulating the miR-150/AIFM2 axis in hepatocellular carcinoma cells

**DOI:** 10.1016/j.heliyon.2024.e37225

**Published:** 2024-08-30

**Authors:** Guangsheng Wang, Yongshan Yao, Jiasheng Xie, Caihong Wen

**Affiliations:** a Department of Gastrointestinal surgery, The First Clinical Medical College of China Three Gorges University, China; b Department of Emergency surgery, The First Clinical Medical College of China Three Gorges University, China; c Department of General surgery, Xiling Community Health Service Center, Xiling District, Yichang City, China; d Department of Medical oncology, The First Clinical Medical College of China Three Gorges University, China

**Keywords:** Ferroptosis, ceRNA, AIFM2, Proliferation

## Abstract

ZNFX1 Antisense RNA 1 (ZFAS1) act as an oncogenic long noncoding RNA in multiple types of cancer. Ferroptosis is an iron-dependent cell death characterized by excessive iron accumulation and lipid peroxidation. However, to date, the functional role and mechanism of ZFAS1 in ferroptosis in hepatocellular carcinoma (HCC) remains largely unknown. The present study revealed that ZFAS1 was upregulated in HCC and upregulation of ZFAS1 indicated poor clinical outcome of HCC patients. Loss- and gain-of-function experiments demonstrated that knockdown of ZFAS1 inhibited HCC cell proliferation and induced ferroptosis, while overexpression of ZFAS1 exerted opposite effects. ZFAS1 enhanced cell proliferation via suppression of ferroptotic death. Mechanistically, ZFAS1 interacted with miR-150 and decreased its expression. AIFM2, the critical ferroptosis protector, was a direct target of ZFAS1/miR-150. ZFAS1 accelerated HCC proliferation and inhibited ferroptosis by the regulation of the miR-150/AIFM2 axis. These discoveries intimate an essential part of ZFAS1/miR-150/AIFM2 in governing HCC ferroptosis, which may provide a promising therapeutic strategy for HCC patients.

## 1 Introduction

Hepatocellular carcinoma (HCC) constitutes the predominant histological subtype of liver cancer, accounting for ninety percent of primary liver cancers. HCC has a high incidence and prevalence worldwide, and is reckoned to be the sixth most prevalent cancer and the fourth predominant cause of cancer-associated deaths throughout the world [1]. There exist several risk elements affiliated with the evolution of HCC, encompassing chronic contagion with hepatitis B or C virus, excessive alcohol consumption, non-alcoholic fatty liver ailment, and exposure to aflatoxins. These risk factors can cause chronic liver damage and inflammation, leading to the development of cirrhosis, which is a major risk factor for HCC [2]. Unfortunately, HCC is often asymptomatic in its early stages, and symptoms may not appear until the cancer has progressed to an advanced stage. This makes early detection and treatment difficult, and many patients are diagnosed when the cancer is already at an advanced stage, leading to poor prognosis and high mortality rates. In addition, HCC is often resistant to chemotherapy and radiation therapy, making it difficult to treat [3]. Therefore, revealing the underlying mechanism of HCC occurrence and development will be crucial for early detection and treatment and improving prognosis in patients with HCC.

Long non-coding RNAs (lncRNAs) constitute a category of non-coding RNAs which are longer than 200 nucleotides in extent and do not encode proteins. In recent years, lncRNAs have been found to play important roles in tumorigenesis and cancer progression [4]. The mechanisms whereby lncRNAs exert their impacts in cancer are variegated and intricate, encompassing epigenetic modulation, transcriptional governance, post-transcriptional ordinance, signaling pathway regulation, and protein interaction [5]. For example, lncRNA HOTAIR can recruit chromatin-modifying enzymes to specific genomic loci, leading to histone modification and gene silencing [6]. lncRNA MALAT1 can interact with the splicing factor SF2/ASF and regulate alternative splicing of target genes [7]. lncRNA H19 can act as a sponge for miR-675, leading to the upregulation of its target genes [8]. ZNFX1 Antisense RNA 1 (ZFAS1) is a lncRNA that is located on chromosome 20q13.13. It was first identified in 2014 and has since been found to be overexpressed in several types of cancer, including colorectal cancer, HCC, and breast cancer. It has been evidenced to foster cancer cell proliferation, migration, incursion, and metastasis, and to restrain apoptosis [[9], [10], [11]]. ZFAS1 has been reported as an oncogenic lncRNA in HCC. ZFAS1 serves as a diagnosis and prognosis biomarker for HCC [12,13]. miR-150 levels are significantly reduced in HCC patients, which suppresses cell migration and invasion [14,15]. ZFAS1 increases ZEB1, MMP14 and MMP16 expression via sponging miR-150, enhancing metastatic capacity of HCC [16]. ZFAS1 activates the extracellular-regulated protein kinases/c-Jun N-terminal kinase (ERK/JNK)/P38 pathway by interacting with midkine (MDK) via miR-624, thereby facilitating the occurrence of HCC [17]. Moreover, upregulation of ZFAS1 expression is closely associated with sorafenib and donafenib resistance in HCC [18,19].

Ferroptosis is a novel form of programmed cell death that is characterized by the accumulation of lipid peroxides and iron-dependent reactive oxygen species (ROS), leading to oxidative damage and cell death. It is different from other forms of cell death and is mainly regulated by the cystine-glutamate antiporter system (system Xc-) and glutathione peroxidase 4 (GPX4). System Xc^−^ maintains GPX4 activity by transporting cystine, and when ferroptosis occurs, GSH depletion leads to GPX4 inactivation, resulting in lipid peroxidation and the generation of ROS, which is the main mechanism of ferroptosis [20,21]. Apoptosis-inducing factor mitochondria-associated 2 (AIFM2), also known as ferroptosis suppressor protein 1 (FSP1), is a NAD(P)H-ubiquinone reductase that plays a crucial role in regulating ferroptosis [22]. AIFM2 is the second most important factor in controlling ferroptosis after GPX4 [23]. AIFM2 is able to efficiently decrease vitamin K to its hydroquinone form, which is a potent antioxidant that can trap free radicals and inhibit the peroxidation of lipids and phospholipids, suggesting that AIFM2 play a key role in protecting cells from oxidative damage and preventing ferroptosis [24]. AIFM2 is conspicuously upregulated in HCC, which is very likely occasioned by DNA hypomethylation and circular RNA circ0060467 [25,26]. Elevated AIFM2 levels are closely associated to poor clinical outcome in HCC patients. The facilitation of metastasis by AIFM2 in HCC is attributed to heightened mitochondrial biogenesis and oxidative phosphorylation through the activating SIRT1/PGC-1α signaling [26]. However, whether lncRNAs are involved in regulating ferroptosis via AIFM2 in HCC is largely unexplored.

It has been shown that ferroptosis contributes to various diseases, including organ damage caused by oxygen deprivation, neurodegeneration, and cancer [27]. Ferroptosis seems to exert a vital role in inhibiting tumors, and actuating ferroptosis might augment cancer therapy, such as through immune checkpoint blockade and radiotherapy [28,29]. For example, cancers with mesenchymal properties or mutations in the E-cadherin-NF2-Hippo pathway are particularly vulnerable to ferroptosis due to changes in redox/iron homeostasis and metabolic processes related to ferroptosis [30]. Therefore, inducing ferroptosis is a promising strategy for treating certain types of cancer with specific genetic characteristics. Recently, ZFAS1 has been found to regulate ferroptosis in diabetic retinopathy, diabetic cardiomyopathy and pulmonary fibrosis [[31], [32], [33]]. Given the liver's pivotal role in iron metabolism, ferroptosis has emerged as a significant player in liver diseases and the carcinogenic process of HCC, offering promising prospects for the eradication of this condition [34]. Increasing evidence revealed that ferroptosis is involved in the proliferation, invasion, and migration of HCC cells, and is intricately linked to drug resistance in HCC [35]. However, the regulatory function of ZFAS1 in ferroptosis in HCC cells remains unknown. In this study, we aim to investigation how ZFAS1 modulates HCC cells resistant to ferroptosis.

## 2 Materials and methods

### 2.1 Patients and specimens

Fresh HCC samples and adjacent noncancerous tissues were collected from 60 patients who initially underwent hepatectomies and were diagnosed with HCC at The First Clinical Medical College of China Three Gorges University. None of the patients had been pretreated with targeted therapy or chemotherapy or radiotherapy before undergoing hepatectomy. Informed consent was procured from all the patients, and the work was sanctioned by the Ethics Committee of the First Clinical Medical College of China Three Gorges University (No. 2022-0156) and executed in accordance with the ethical precepts of the Declaration of Helsinki.

### 2.2 Cell culture and transfection

A normal liver cell line (THLE-2) was cultured in BEGM (Bronchial Epithelial Cell Growth Medium) containing 10 % fetal bovine serum (FBS), and six HCC cell lines (Hep3B, Huh7, PLC/PRF/5, SNU182, and MHCC-97h) were cultured in DMEM medium containing 10 % FBS. All cells were cultured in in 5 % CO_2_ at 37 °C, authenticated by short tandem repeat (STR) analysis and verified to be mycoplasma-exempt. Lipofectamine 3000 (Invitrogen) was employed for transfection in accordance with the product directives. In brief, a total of 1 × 10^6^ cells per well were seeded onto six-well plates, diluted in 250 μl of Opti-MEM for both negative control (NC) and miR-150 mimics or inhibitor. The transfection reagent was delicately inverted and diluted in 250 μl of Opti-MEM, followed by the addition of 5 μl of Lipofectamine 3000, allowing the mixture to rest for 5 min at ambient temperature. Subsequently, the transfection reagent was combined with the NC/miRNA dilution, and the amalgamation was left to incubate for 20 min at room temperature. The transfection complex was introduced into each well of the six-well plate and gently swirled before and after agitation. Transfection was concluded following 48 h of incubation.

### 2.3 Construction of stable cells

ZFAS1 overexpression and knockdown lentiviruses were constructed and then infected into Huh7 and Hep3B cells, respectively. Stable cells were picked out by means of puromycin for a fortnight. The target sequence of ZFAS1 shRNA was used as previous study described [16] and listed as follow: shZFAS1: TCCAAAATCCATTCTGTACCC.

### 2.4 CCK-8 assay

Cell proliferation and survival was examined by using CCK-8 assay. 3 × 10^3^ cells were seeded on 96-well plate in triplicate. The cell numbers were determined by Cell Counting Kit-8 (CCK-8) assay at indicated time point. 10 μL CCK-8 reagent was added into cells, and then incubated for 1 h. The absorbance at 450 nm was The absorbance at 450 nm was measured using microplate reader (Thermo).

### 2.5 Colony formation assay

1 × 10^3^ cells were seeded on 6-well plate in triplicate and then cultured for 7–10 days. The cells were immobilized by paraformaldehyde and tinctured by crystal violet. The clones fashioned by diverse cells were photographed and enumerated.

### 2.6 Western blot

The cell lysates were prepared using lysis buffer (50 mM Tris-HCl, pH 7.5, 150 mM NaCl, 1 % NP-40, 0.5 % Na-deoxycholate) containing protease inhibitor cocktail (Roche). The protein concentration was determined using the Bradford method (Bio-Rad Laboratories). SDS-PAGE electrophoresis was used to resolve 20–100 μg of protein, which was then electroblotted onto PVDF membrane (Millipore). Primary antibodies were incubated with the membranes at 4 °C overnight, followed by secondary antibodies for 2 h at room temperature. The antibodies used was shown as follow: anti-AIFM2 (Cell Signaling, #24972), anti-GAPDH (Cell Signaling, #5174). The Western Blotting Chemiluminescence Luminol Reagent (Millipore) was used to detect antibody-antigen complexes. The original images of Western blot were shown in Supplemental Fig. 1.

### 2.7 RNA isolation and qRT-PCR

Fresh GC tissues and cell lines were subjected to RNA extraction using the Trizol reagent (Takara, Japan). qRT-PCR was performed using the LightCycler 96 system (RD, Basel, Switzerland) and SYBR Green Pro Taq HS Premix (AG, China). The primer sequences used for qRT-PCR were listed as follows: ZFAS1-forward: ACCAGTTCCACAAGGTTACTG, ZFAS1-reverse: CTTTATGCAGGTAGGCAGTTAGA; AIFM2-forward: GGCCAACATCGTCAACTCT, AIFM2-reverse: CCCACATAGAAGCCACTGATT.

### 2.8 RNA immunoprecipitation (RIP)

The RIP assay was carried out by utilizing the Magna RIP™ RNA-Binding Protein Immunoprecipitation Kit (Millipore, Bedford, MA). Briefly, cells were disrupted in a lysis buffer encompassing a protease inhibitor cocktail and RNase inhibitor. Magnetic beads were pre-incubated with either an anti-AGO2 antibody or anti-rabbit IgG for 30 min at ambient temperature. Thereafter, lysates were immunoprecipitated with the beads at 4 °C throughout the night. RNA was extracted from the RNA-protein complexes adhered to the beads and subjected to analysis by qRT-PCR.

### 2.9 MS2-RIP assay

MS2-RIP was carried as a previous study described. In brief, cells were co-transfected with pcDNA3.1-MS2, pcDNA3.1-MS2-ZFAS1, pcDNA3.1-MS2-ZFAS1-mut and pMS2-GFP (Addgene). After 48 h, an RIP experiment was performed using a GFP antibody (Abcam) and the Magna RIP™ RNA-Binding Protein Immunoprecipitation Kit (Millipore, Bedford, MA) according to the product instructions.

### 2.10 RNA pull-down assay

The method of RNA pull-down assays was utilized, following previously described protocols. RNAs were marked with biotin by means of the Biotin RNA Labeling Mix (Roche) and transcribed *in vitro* with T7 RNA polymerase (Roche). The resultant RNA was dealt with by RNase-free DNase I (Roche) and purified via the RNeasy® Plus Mini Kit (Qiagen). Thereafter, the biotinylated RNAs were mingled with huh7 cell lysates and incubated. Streptavidin agarose beads (Life Technologies) were appended to each binding reaction and incubated at ambient temperature for 1 h. Subsequently, the beads were subjected to boiling in SDS buffer, and the eluted proteins were ascertained by means of Western blot analysis.

### 2.11 Luciferase reporter assays

ZFAS1 or AIFM2 3′UTR was inserted into pmirGLO plasmid. Cells were cultured in 24-well plate, and transfected with reporter plasmid and control or miR-150 mimics or inhibitor. 48 h thereafter, the cells were lysed, and the activities of firefly and Renilla luciferase were ascertained by means of the Dual-Luciferase® Reporter Assay System (Promega).

### 2.12 Measurement of GSH, ROS, Fe^2+^ and MDA

The GSH, ROS, Fe^2+^ and MDA levels were measured using Reduced Glutathione (GSH) Colorimetric Assay Kit, Reactive Oxygen Species (ROS) Fluorometric Assay Kit (Elabscience), Cell Ferrous Iron Colorimetric Assay Kit (Elabscience) and Malondialdehyde (MDA) Colorimetric Assay Kit (Elabscience) according to the product instructions, respectively. Then, the GSH concentration can be determined by assessing the OD value at 420 nm, Fe^2+^ concentration can be calculated by measuring the OD value at 593 nm, MDA concentration can be determined by assessing the OD value at 532 nm with a microplate reader, and ROS levels were quantified using a fluorescence microplate reader (excitation wavelength 485–515 nm, emission wavelength 510–550 nm).

### 2.13 Statistical analysis

SPSS software (Abbott Laboratories, Chicago, IL) was used for all statistical analyses. Survival curves were generated using Kaplan-Meier and log-rank tests. The Mann-Whitney *U* test was employed to examine the association between lincRNA-ZFAS1 expression and clinicopathological characteristics. The difference among different groups were analyzed using Student's t-test or multi-way classification ANOVA tests. The correlation between ZFAS1 and AIFM2 was evaluated using Spearman rank correlation. A p-value of less than 0.05 was considered statistically significant.

## 3 Results

### 3.1 Overexpression of ZFAS1 indicates a worse prognosis and promotes the proliferation of HCC cells

**Fig. 1:**
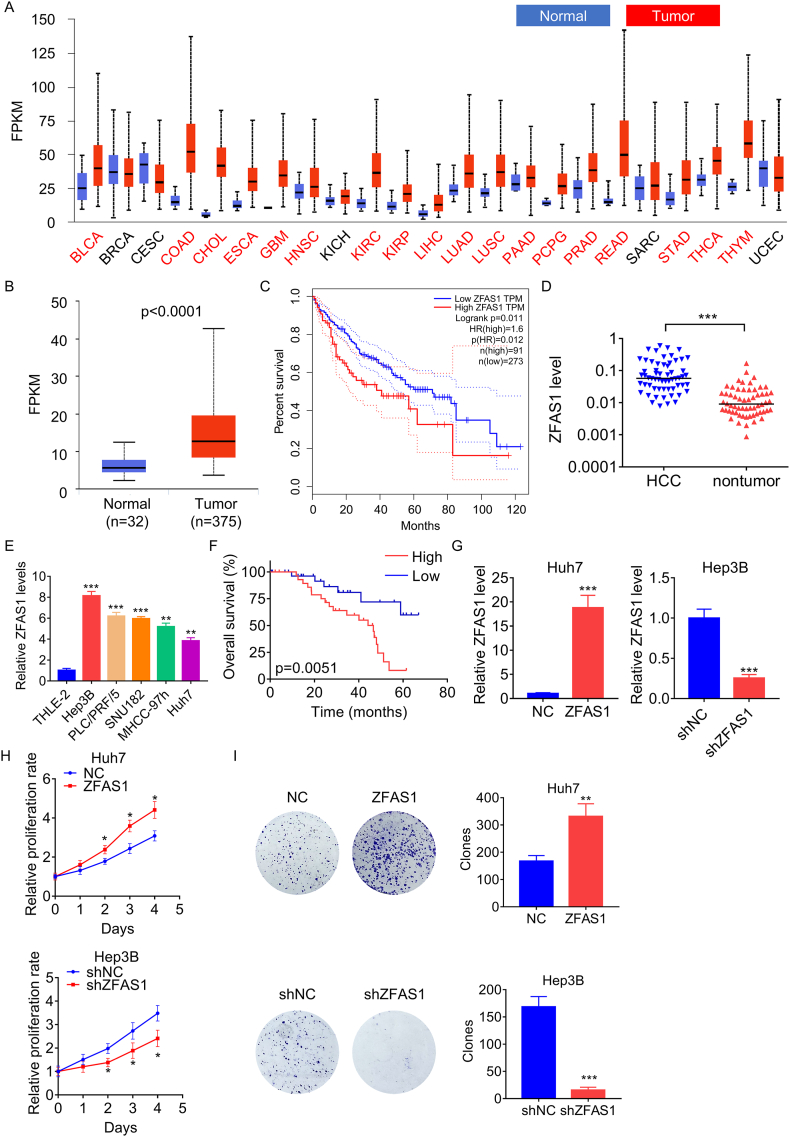
ZFAS1 is overexpressed in HCC and promotes proliferation. A. The ZFAS1 expression in different tumor and normal tissues from TCGA database. B. The difference of ZFAS1 expression between HCC and normal liver tissues from TCGA database. C. The survival analysis of HCC patients with high and low ZFAS1 based on TCGA database. D. qRT-PCR analysis of ZFAS1 levels in 60 pairs of HCC and corresponding adjacent nontumor tissues. E. qRT-PCR analysis of ZFAS1 levels in a normal liver cell line and different HCC cell lines. F. Kaplan‐Meier survival curves for HCC patients with high and low ZFAS1. G. The ZFAS1 was overexpressed and knocked down in Huh7 and Hep3B cells, respectively. The overexpression and knockdown efficacy was validated using qRT-PCR. H. The effect of ZFAS1 overexpression or knockdown on cellular proliferation was measured using CCK-8 assays. I. The clone formed by HCC cells with ZFAS1 overexpression or knockdown. *p < 0.05, **p < 0.01, ***p < 0.001.

The expression levels of ZFAS1 were evaluated in 23 primary tumor types using The Cancer Genome Atlas (TCGA) dataset. By utilizing the UALCAN online platform (https://ualcan.path.uab.edu/index.html), it was observed that ZFAS1 levels exhibited a significant elevation compared to those in normal tissues across 18 types of tumors (Fig. 1A). In particular, ZFAS1 expression was markedly elevated in HCC tumor tissues compared to normal tissues (Fig. 1B). Furthermore, Kaplan-Meier survival analysis disclosed that patients possessing high expression levels of ZFAS1 had a shorter overall survival in comparison to those with low expression levels of ZFAS1 (Fig. 1C). To validate these findings, ZFAS1 expression was measured using qRT-PCR in 60 pairs of HCC and corresponding adjacent nontumor tissues, which showed that ZFAS1 was significantly upregulated in HCC tissues compared to nontumor tissues (Fig. 1D). Similarly, HCC cell lines (Hep3B, Huh7, PLC/PRF/5, SNU182, and MHCC-97h) exhibited elevated ZFAS1 expression levels compared to a normal liver cell line (THLE-2) (Fig. 1E). Moreover, Kaplan-Meier survival analysis succeeded by a log-rank test affirmed that HCC patients possessing higher ZFAS1 levels exhibited a poorer overall survival rate than those with lower ZFAS1 levels (Fig. 1F).

In order to explore the biological function of ZFAS1 in HCC cells, Huh7 cells possessing relatively low ZFAS1 expression and Hep3B cells with relatively high ZFAS1 expression were chosen to establish stable cell lines with ZFAS1 overexpression and knockdown respectively. The overexpression and knockdown efficacy were validated using qRT-PCR (Fig. 1G). Results from CCK-8 showed that overexpression of ZFAS1 promoted the proliferation abilities of Hep3B cells, while knockdown of ZFAS1 had the opposite effect in Huh7 cells (Fig. 1H). Furthermore, the colony formation assays revealed that the quantity and dimensions of colonies generated by Hep3B cells overexpressing ZFAS1 were markedly greater compared to those formed by the control cells. Conversely, depletion of ZFAS1 reversed this impact in Huh7 cells (Fig. 1I). These findings suggest that ZFAS1 functions as an oncogene in HCC cells.

### 3.2 ZFAS1 enhances HCC cell proliferation by inhibiting ferroptosis

**Fig. 2:**
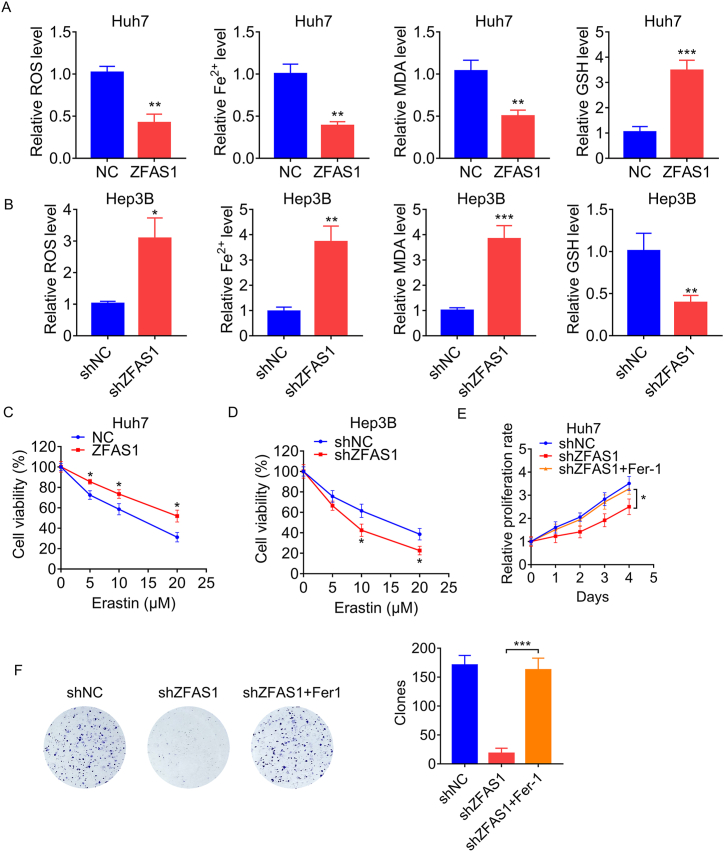
ZFAS1 regulates ferroptosis. A. The ROS, Fe^2+^, MDA and GSH levels in control and ZFAS1-overexpressing Huh7 cells. B. The ROS, Fe^2+^, MDA and GSH levels in control and ZFAS1-silencing Hep3B cells. C. Control and ZFAS1-overexpressing Huh7 cells were treated with indicated concentration of erastin for 48 h. Then, cell viability was detected using CCK-8 assay. D. Control and ZFAS1-silencing Hep3B cells were treated with indicated concentration of erastin for 48 h. Then, cell viability was detected using CCK-8 assay. E-F, E-F. ZFAS1-silencing Hep3B cells were treated with 2 μM ferrostatin-1 (Fer-1). Then, the proliferation was detected by CCK-8 (E) and colony formation (F) assays. *p < 0.05, **p < 0.01, ***p < 0.001.

We assay the influence of ZFAS1 upon ferroptosis in HCC. GSH, ROS, and Fe^2+^ are indispensable for the ferroptosis procedure, and malondialdehyde (MDA) is a significant end product of ROS. Therefore, we measured their concentrations in Huh7 cells stably expressing ZFAS1. As evidenced by enhanced GSH levels and decreased accumulation of ROS, Fe^2+^ and MDA, ferroptosis was inhibited by ZFAS1 overexpression (Fig. 2A). Through the same detection, the reduced GSH levels and increased levels of ROS, Fe^2+^ and MDA suggested that knockdown of ZFAS1 expression promoted ferroptosis in Hep3B cells (Fig. 2B). To determine the effect of ZFAS1 on erastin-mediated (an inducer of ferroptosis) ferroptosis, CCK-8 assays was performed and indicated that upregulation of ZFAS1 significantly increased the survival of Huh7 cells treated with erastin compared to the control cells (Fig. 2C). Conversely, depletion of ZFAS1 sensitizes Hep3B cells to erastin-induced ferroptosis (Fig. 2D). Then, we detected whether ZFAS1 promotes cell proliferation via inhibition of ferroptosis. Ferrostatin-1 (an inhibitor of ferroptosis, Fer-1) was added into the ZFAS1-silencing Hep3B cells. The results of CCK-8 and colony formation assays confirmed that Fer-1 could block the ZFAS1-silencing-induced proliferation suppression (Fig. 2E and F). Taken together, our data suggest that ZFAS1 enhances HCC cell proliferation by inhibiting ferroptosis.

### 3.3 ZFAS1 acts as a sponge for miR-150

**Fig. 3:**
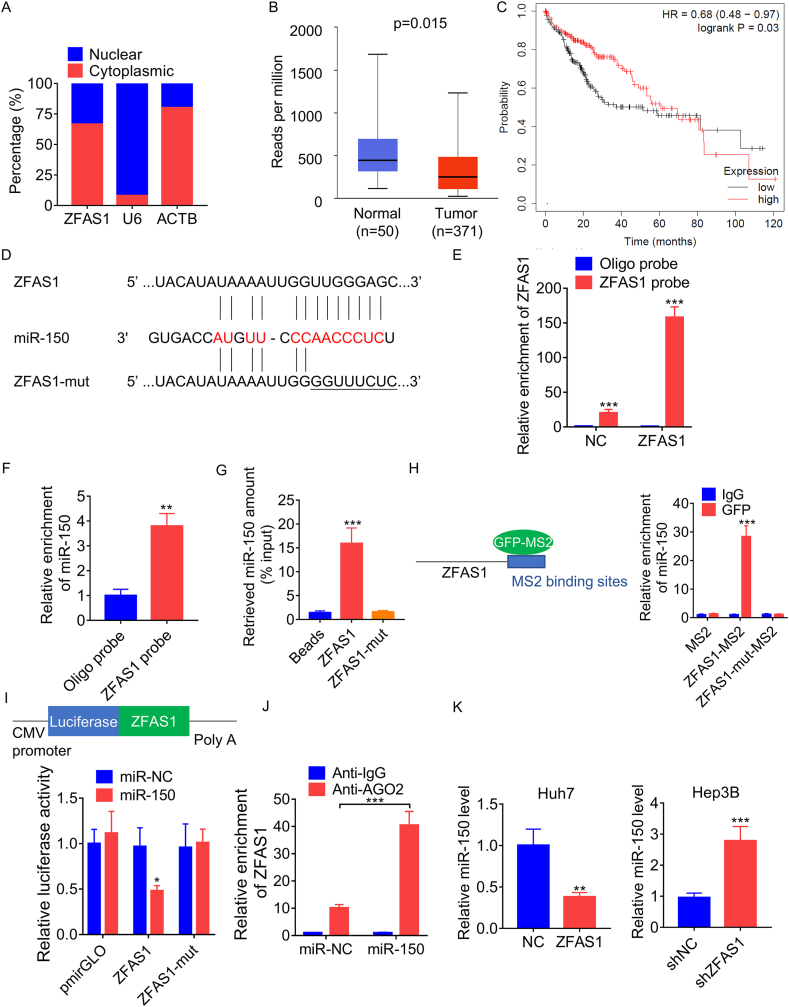
ZFAS1 associates with miR-150. A. The subcellular distribution of ZFAS1 was detected using nuclear-cytoplasmic isolation followed by qRT-PCR. B. The difference of miR-150 expression between HCC and normal liver tissues from TCGA database. C. The survival analysis of HCC patients with high and low miR-150 based on TCGA database. D. The putative binding sites between miR-150 and ZFAS1. E. Lysates prepared from Huh7 cells with or without ZFAS1 overexpression were subject to RNA pull-down assay, and the efficiency was confirmed by qRT-PCR. F. The relative levels of miR-150 pulled down by oligo or ZFAS1 probe in the Huh7 cell lysates were examined by qRT-PCR. G. The miR-150 pulled down by biotin-labeled ZFAS1 in Huh7 cell lysates was assessed by qRT-PCR. H. The MS2-RIP followed by qRT-PCR was carried out to detect the interaction between wild-type or mutant ZFAS1 and miR-150. I. The luciferase reporter containing wild-type or mutant ZFAS1 was cotransfected with miR-NC or miR-150 into Hep3B cells. Then, the luciferase activity was measured. J. Anti-Ago2 RIP assay was conducted in Hep3B cells after transfection with miR-NC or miR-150 mimics, followed by qRT-PCR analysis to detect the expression levels of ZFAS1. K. The relative miR-150 levels in Huh7 cells with ZFAS1 overexpression and Hep3B cells with ZFAS1 knockdown. *p < 0.05, **p < 0.01, ***p < 0.001.

To investigate the mechanism of ZFAS1 in HCC cells, we conducted a nuclear-cytoplasmic fractionation assay and found that ZFAS1 mainly localized in the cytoplasm (Fig. 3A). Cytoplasmic lncRNAs is able to act as miRNA sponges to regulate the expression of target mRNAs by relieving miRNA inhibitory effects. Recently, bioinformatics analysis predicted that ZFAS1/miR-150 regulatory axis had a high diagnostic and prognostic value for HCC patients [36]. Moreover, it has been reported that miR-150 is involved in regulating ferroptosis in colorectal cancer cells [37]. The analysis of TCGA database showed that miR-150 levels were markedly decreased in HCC tissues and its downregulation indicated poor clinical outcome in HCC patients (Fig. 3B and C). Therefore, it was hypothesized that ZFAS1 might serve as a miRNA sponge for miR-150 in HCC. Using the LncBook and miRcode online tool, we found that ZFAS1 contains the binding site of miR-150 (Fig. 3D). To validate the interaction between ZFAS1 and miR-150, RNA pull-down assays using a specific biotin-labeled probe against ZFAS1 and *in vitro* transcribed biotin-labeled ZFAS1 were carried out, respectively. The pull-down efficacy was conspicuously augmented subsequent to the overexpression of ZFAS1, and miR-150 was prominently enriched by the biotinylated ZFAS1 probe (Fig. 3E and F). Additionally, we found that miR-150 could be significantly pulled down by the wild-type ZFAS1, but not by the ZFAS1 with mutation in miR-150 binding sites (ZFAS1-mut) (Fig. 3G). To further corroborate the interplay between ZFAS1 and miR-150, we undertook an MS2-RIP assay to draw down endogenous microRNAs affiliated with ZFAS1. Our findings manifested that miR-150 could be conspicuously enriched by wild-type ZFAS1 in contrast to the vacant vector (MS2), IgG, and ZFAS1-mut (Fig. 3H). Additionally, we constructed luciferase reporters containing wild-type and mutant ZFAS1 and conducted a dual-luciferase reporter assay. The results revealed that the luciferase activity of the wild-type ZFAS1 reporter could be significantly suppressed by miR-150 mimics, in contrast to the mutant reporter vector (Fig. 3I).

miRNAs attach to their target RNAs for degradation or translational repression principally in an AGO2-dependent mode. To explore whether ZFAS1 binds to miR-150-5p in an AGO2-dependent manner, we carried out an anti-AGO2 RIP assay. Our results showed that ZFAS1 and miR-150 were efficiently enriched by anti-AGO2 antibodies, but not by the negative control IgG antibodies. Furthermore, both ZFAS1 and miR-150 were significantly enriched in Huh7 and Hep3B cells with miR-150 overexpression compared to the negative control group (Fig. 3J).

Subsequently, we ascertained the impact of ZFAS1 upon miR-150 expression. In accordance with the qRT-PCR assays, the depletion of ZFAS1 led to an augmentation in miR-150 expression in Hep3B cells when contrasted with the control cells, while the enhancement of ZFAS1 repressed the level of miR-150 in huh7 cells (Fig. 3K). In sum, these discoveries signify that ZFAS1 serves as a sponge for miR-150 in HCC.

### 3.4 ZFAS1 positively regulates AIFM2 expression via sponging miR-150

**Fig. 4:**
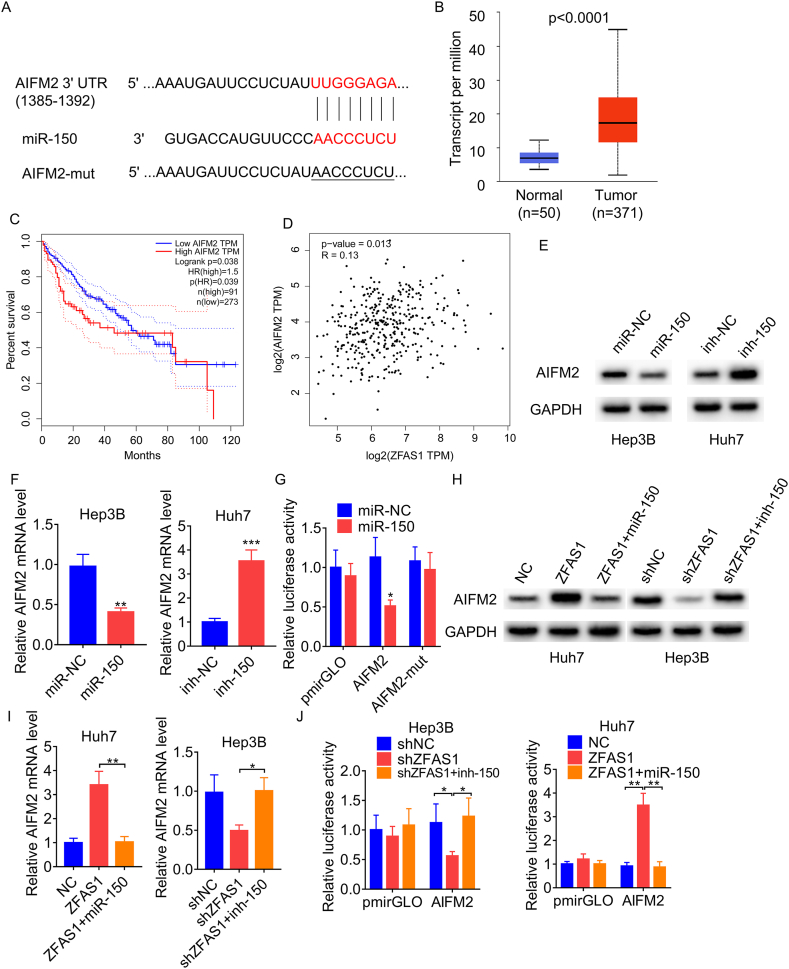
ZFAS1 regulates AIFM2 via miR-150. A. The putative binding sites between miR-150 and AIFM2 3′UTR. B. The difference of AIFM2 mRNA expression between HCC and normal liver tissues from TCGA database. C. The survival analysis of HCC patients with high and low AIFM2 based on TCGA database. D. The correlation analysis between ZFAS1 and AIFM2 expression based on TCGA database. E. The effect of miR-150 overexpression or knockdown on AIFM2 protein level was detected using Western blot in Hep3B and Huh7 cells, respectively. F. The effect of miR-150 overexpression or knockdown on AIFM2 mRNA level was detected using qRT-PCR in Hep3B and Huh7 cells, respectively. G. The luciferase reporter containing wild-type or mutant AIFM2 3′UTR was cotransfected with miR-NC or miR-150 into Hep3B cells. Then, the luciferase activity was measured. H. The effect of miR-150 overexpression or knockdown on ZFAS1-changed AIFM2 protein level was tested using Western blot. I. The effect of miR-150 overexpression or knockdown on ZFAS1-changed AIFM2 mRNA level was tested using qRT-PCR. J. The effect of miR-150 overexpression or knockdown on ZFAS1-changed activity of AIFM2 reporter was tested using luciferase reporter assay. *p < 0.05, **p < 0.01, ***p < 0.001.

We further explored which downstream molecules was modulated by the ZFAS1/miR-150 axis to influence the ferroptosis of HCC. LncRNAs can function as competitive endogenous RNAs to associate with miRNAs and then terminate the suppressive effects on their targets. The genes associated with ferroptosis encompass CDKN1A, CISD1, LPCAT3, NCOA4, EMC2, GPX4, HSPA5, HSPB1, MT1G, NFE2L2, SAT1, SLC1A5, SLC7A11, ACSL4, ALOX15, FANCD2, FDFT1, ATL1, ATP5MC3, CARS1, CS, DPP4, GLS2, RPL8, TFRC, and AIFM2. Through Targetscan online tool predication, we found that only AIFM2 could be predicted as the target of miR-150 (Fig. 4A), which play a critical role in inhibiting ferroptosis [24]. The analysis of TCGA database revealed that AIFM2 was significantly upregulated in HCC tissues and associated with the overall survival of HCC patients (Fig. 4B and C). Furthermore, ZFAS1 was positively associated with AIFM2 in HCC tissues (Fig. 4D). In light of these discoveries, we surmised that AIFM2 might be implicated in the ZFAS1/miR-150 axis-governed ferroptosis.

To investigate whether AIFM2 is a direct target of miR-150, we performed qRT-PCR and Western blot assays and found that transfection of miR-150 mimics obviously decreased both mRNA and protein levels of AIFM2 (Fig. 4E and F). Additionally, luciferase reporters containing wild-type and mutant AIFM2 3′UTR were constructed, and the findings of luciferase reporter assays showed that the miR-150 mimics significantly decreased the luciferase activity of the wild-type AIFM2 reporter, but did not show an obvious effect on its mutant (Fig. 4G).

Next, we investigated whether ZFAS1 functions as a ceRNA of AIFM2 against miR-150. ZFAS1 upregulated both mRNA and protein levels of AIFM2, which could be abolished by transfection of miR-150 mimics. Conversely, AIFM2 expression was markedly suppressed by knockdown of ZFAS1, while inhibition of miR-150 reversed this effect (Fig. 4H and I). To verify whether this perceived effect is contingent upon the regulation of the AIFM2 3′UTR, luciferase plasmids (pmirGLO and pmirGLO-AIFM2) were introduced into cells. The silencing of ZFAS1 diminished the luciferase activity of pmirGLO-AIFM2, which was restored by the inhibition of miR-150. Conversely, the overexpression of ZFAS1 augmented the luciferase activity of pmirGLO-AIFM2, and the overexpression of miR-150 nullified this upregulation (Fig. 4J). These findings imply that ZFAS1 regulates AIFM2 by means of competitively binding miR-150.

### 3.5 ZFAS1 regulates proliferation and ferroptosis by modulating miR-150/AIFM2 axis

**Fig. 5:**
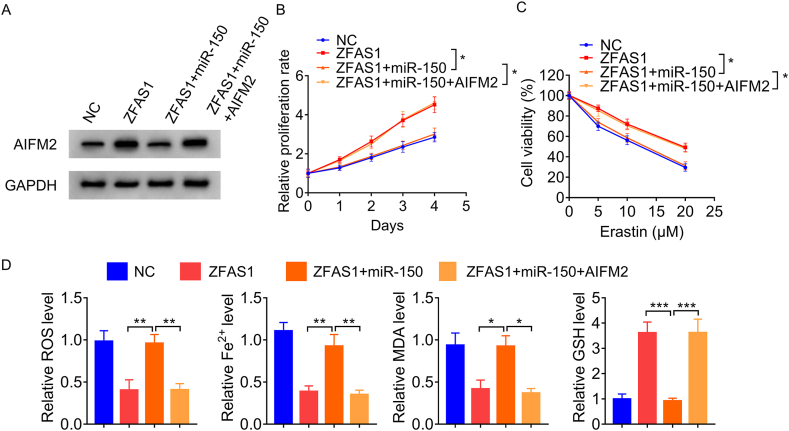
ZFAS1 regulates proliferation and ferroptosis via miR-150/AIFM2 axis. A. ZFAS1-overexpressing Huh7 cells were transfected with miR-150 mimics alone or in combination with AIFM2. Then, AIFM2 expression was tested using Western blot. B. CCK-8 assays evaluated the effect of the ZFAS1/miR-150/AIFM2 axis on cell proliferation in Huh7 cells. C. CCK-8 assays evaluated the effect of the ZFAS1/miR-150/AIFM2 axis on erastin-indued cell death in Huh7 cells. D. Effects of the ZFAS1/miR-150/AIFM2 axis on ROS, Fe^2+^, MDA and GSH levels Huh7 cells. *p < 0.05, **p < 0.01, ***p < 0.001.

Finally, rescue experiments were carried out to ascertain whether ZFAS1 regulates proliferation and ferroptosis of HCC ells by competitively binding to miR-150 and subsequently increasing AIFM2 expression. ZFAS1-overexpressing Huh7 cells were transfected with miR-150 mimics singly or in conjunction with AIFM2 (Fig. 5A).The results of CCK-8 assay demonstrated that the increased proliferation of ZFAS1-overexpresing Huh7 cells was effectively reversed by miR-150 mimics; co-transfection of AIFM2 could notably abolish these rescue effects of miR-150 on ZFAS1-overexpresing Huh7 cells (Fig. 5B). Moreover, overexpression of miR-150 abolished increased GSH levels, decreased ROS, Fe^2+^ and MDA levels and a decreased percentage of cell death upon erastin induction following ZFAS1 overexpression, while the restoration of AIFM2 attenuated these miR-150-mediated effects (Fig. 5C and D). Taken together, these data suggested that ZFAS1/miR-150/AIFM2 axis play an important role in regulating HCC cell proliferation and ferroptosis.

## 4 Discussion

Increasing evidences have shown that ZFAS1 promote the initiation and development of multiple human cancers. For example, ZFAS1 facilitates colorectal cancer cell proliferation, migration, and increased cell apoptosis. ZFAS1 associates with NOP58, promoting the small nucleolar RNA-mediated 2′-O-methylation and subsequently regulating the RNA stability and translational activity of their downstream targets [38]. ZFAS1 facilitates the advancement and metastasis of colorectal cancer by serving as a competing endogenous RNA of miR-144 to modulate EZH2 expression [39]. In HCC, it was reported that upregulation of ZFAS1 indicates poor prognosis [16], which is consistent with our findings in this study. ZFAS1 has been found to enhance the proliferation and metastasis abilities of HCC, and increase the resistance of sorafenib treatment as well [16,18,40]. Ferroptosis play important roles in regulating cell survival and antidrug resistance in HCC. For example, stimulation of the p62-Keap1-Nrf2 pathway impedes Nrf2 degradation, thereby shielding HCC cells from ferroptosis. Inhibition of Nrf2 expression, either genetically or pharmacologically, has the potential to enhance sorafenib's antitumor efficacy in both *in vitro* and tumor xenograft models [41]. As revealed by recent research, ZFAS1 is able to induce ferroptotic death. In diabetic retinopathy, ZFAS1 facilitates endothelial ferroptosis via miR-7-5p/ACSL4 axis [33]. In diabetic cardiomyopathy, ZFAS1 induces ferroptosis by sponging miR-150-5p and activates CCND2 expression [32]. ZFAS1 facilitates the transition of lung fibroblasts to myofibroblasts and ferroptosis by serving as a ceRNA via the miR-150-5p/SLC38A1 axis in the course of pulmonary fibrosis [31]. However, it is unclear whether ZFAS1 is involved in the ferroptosis of cancer cells. Here, our data demonstrated that ZFAS1 exerts a suppressive effect on ferroptosis, as evidenced by accumulation of ROS, Fe^2+^ and MDA and decrease of GSH level upon ZFAS1 overexpression. These findings suggest that ZFAS1 has a promotive or inhibitory function in ferroptosis, which may depend on different kind of diseases.

Biochemical functions of RNA are closely related to their subcellular distribution. Cytoplasmic lncRNAs can act as microRNA sponge, and inhibit the degradation or translational repression of target mRNAs which is mediated by microRNAs [42]. Previous studies have demonstrated that ZFAS1 can associate with some microRNAs, including miR-153, miR-124, miR-190a and miR-150 [[43], [44], [45]]. Here, ZFAS1 was found to mainly distribute in the cytoplasm of HCC cells and bond with the AGO2 protein, further confirming that ZFAS1 exert its oncogenic function via this way. Through RNA pull-down, MS2-RIP, and luciferase reporter experiments, miR-150 was further confirmed as a direct target of ZFAS1. ZFAS1 associated with miR-150 and suppressed its expression. Previous studies have implicated miR-150 in the pathogenesis of multiple tumors [[46], [47], [48]]. Additionally, there have been reports indicating the involvement of miR-150 in modulating ferroptosis by targeting c-Myb in cancer cells [37]. Our results demonstrated that the miR-150 expression was decreased in HCC and its downregulation indicated poor prognosis of HCC patients. Moreover, rescue experiments verified that the restoration of miR-150 expression could markedly nullify the ZFAS1-mediated proliferation facilitation and ferroptosis suppression.

It has been reported that the downstream genes of ZAFS1/miR-150 includes VEGFA, ST6GAL1, ZEB1 and RAB9A in cancer cells [16,[49], [50], [51]]. However, the objects of ZFAS1/miR-150 in governing ferroptosis remain unbeknownst. Hereinafter, AIFM2 was recognized as a novel objective of miR-150. ZFAS1 augmented AIFM2 expression, which was nullified by miR-150. Furthermore, rescue experiments manifested that ZFAS1 advanced cell proliferation and repressed ferroptosis through AIFM2. The affirmative correlation between ZFAS1 and AIFM2 in HCC tissues further attested that AIFM2 was a target of ZFAS1/miR-150. In parallel with GPX4, AIFM2 serves as an alternative inhibitor of ferroptosis. AIFM2 effects the NADH-dependent reduction of CoQ on the plasma membrane, thereby inhibiting phospholipid peroxidation and ferroptosis [52]. The regulatory relationship between lncRNA and AIFM2 remains largely unknown. LncRNA lncFAL has been disclosed to diminish the vulnerability to ferroptosis by directly binding to AIFM2 and competitively nullifying the Trim69-dependent AIFM2 polyubiquitination degradation [53]. Here, we demonstrated a new regulatory mechanism of AIFM2 expression via ZFAS1/miR-150 axis.

There exist certain constraints in our studies. Firstly, our discoveries have unveiled the pivotal role of the ZFAS1/miR-150/AIFM2 axis in inhibiting ferroptosis in HCC cells under *in vitro* conditions. Subsequent validation through an *in vivo* animal study is imperative to fortify our conclusions. Secondly, the correlation among ZFAS1, miR-150 and AIFM2 expression in HCC tissues were analyzed in TCGA database, which should be further validated in clinical samples. Furthermore, ZFAS1/miR-150 regulatory axis has been reported to show a high diagnostic and prognostic value for HCC patients [36]. In light of this, it is conceivable that the ZFAS1/miR-150/AIFM2 axis could serve as a more robust diagnostic and prognostic biomarker for HCC patients, surpassing the utility of ZFAS1 alone or the ZFAS1/miR-150 axis. Thirdly, we did not explore the therapeutic potential of targeting ZFAS1 in HCC. NcRNA inhibitors encompass short hairpin RNAs (shRNAs), antisense anti-oligonucleotides (ASOs), siRNAs, antagomirs, miRNA sponges, circRNA sponges, and CRISPR/Cas9-based genome editing [54]. ASOs signify single-stranded RNA molecules which adhere to complementary RNA sequences with precisely matched base pairing to hinder and impede their functionality, thereby resulting in their degradation via RNAse-H-mediated cleavage. Furthermore, in preclinical *in vivo* investigations, ASOs display targeted and efficacious reduction in lncRNA levels [55]. The utilization of ASOs to target ZFAS1 may hold promise for HCC therapy. Fourthly, ferroptosis is mainly controlled by NAD(P)H-AIFM2-CoQ10 and NAD(P)H-GSH-GPx4 pathways. Our current findings have only unveiled the involvement of ZFAS1 in AIFM2 expression. The question of whether ZFAS1 modulates ferroptosis through the NAD(P)H-GSH-GPx4 pathway remains shrouded in ambiguity. Lastly, it may be imperative to explore the potential downstream targets of the ZFAS1/miR-150/AIFM2 axis. For instance, AIFM2 catalyzes the regeneration of CoQ10 using NAD(P)H to suppress phospholipid peroxidation and ferroptosis [22]. Furthermore, AIFM2 triggers SIRT1/PGC-1α signaling to amplify mitochondrial biogenesis and oxidative phosphorylation [26]. Whether these downstream effectors are under the regulation of the ZFAS1/miR-150/AIFM2 axis warrants further investigation.

## Summary

**Fig. 6:**
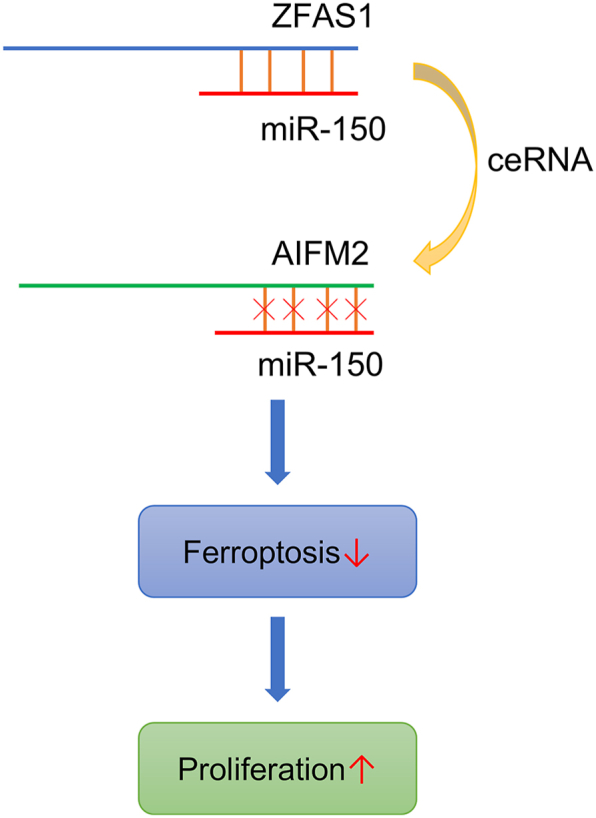
Schematic figure showing the crucial roles of ZFAS1/miR-150/AIFM2 axis in HCC.

In sum, we identified the role and molecular mechanism of ZFAS1 in regulating ferroptosis via miR-150/AIFM2 axis. The overexpression of ZFAS1 betokens the unfavorable prognosis of HCC patients. ZFAS1 furthers HCC cell proliferation by inhibiting ferroptosis. Mechanistically, ZFAS1 associates with and represses miR-150, subsequently increasing AIFM2 expression (Fig. 6). Our findings suggest that ZFAS1/miR-150/AIFM2 axis may be a potential target for HCC patients.

## Consent for publication

Not applicable.

## Data availability statement

The datasets used during this research are available. Data included in article/supp. material/referenced in article. Data associated with our study has not been deposited into a publicly available repository.

## Ethics approval and consent to participate

The present study was approved by the Ethics Committee of the First Clinical Medical College of China Three Gorges University and carried out in accordance with the World Medical Association Declaration of Helsinki.

## CRediT authorship contribution statement

**Guangsheng Wang:** Project administration, Investigation. **Yongshan Yao:** Investigation, Data curation. **Jiasheng Xie:** Investigation. **Caihong Wen:** Investigation.

## Declaration of competing interest

The authors declare that they have no known competing financial interests or personal relationships that could have appeared to influence the work reported in this paper.

## Figures and Tables

**figs1:**